# Lentiviral Vectors to Probe and Manipulate the Wnt Signaling Pathway

**DOI:** 10.1371/journal.pone.0009370

**Published:** 2010-02-23

**Authors:** Christophe Fuerer, Roel Nusse

**Affiliations:** Howard Hughes Medical Institute, Department of Developmental Biology, Stanford University School of Medicine, Stanford, California, United States of America; New Mexico State University, United States of America

## Abstract

**Background:**

The Wnt signaling pathway plays key roles in development, adult tissue homeostasis and stem cell maintenance. Further understanding of the function of Wnt signaling in specific cell types could benefit from lentiviral vectors expressing reporters for the Wnt pathway or vectors interfering with signaling.

**Methodology/Principal Findings:**

We have developed a set of fluorescent and luminescent lentiviral vectors that report Wnt signaling activity and discriminate between negative and uninfected cells. These vectors possess a 7xTcf-eGFP or 7xTcf-FFluc (Firefly Luciferase) reporter cassette followed by either an SV40-mCherry or SV40-Puro^R^ (puromycin N-acetyltransferase) selection cassette. We have also constructed a vector that allows drug-based selection of cells with activated Wnt signaling by placing Puro^R^ under the control of the 7xTcf promoter. Lastly, we have expressed dominant-negative Tcf4 (dnTcf4) or constitutively active beta-catenin (β-catenin^4A^) from the hEF1α promoter in a SV40-Puro^R^ or SV40-mCherry backbone to create vectors that inhibit or activate the Wnt signaling pathway. These vectors will be made available to the scientific community through Addgene.

**Conclusions:**

These novel lentiviruses are efficient tools to probe and manipulate Wnt signaling. The use of a selection cassette in Wnt-reporter viruses enables discriminating between uninfected and non-responsive cells, an important requirement for experiments where selection of clones is not possible. The use of a chemiluminescent readout enables quantification of signaling. Finally, selectable vectors can be used to either inhibit or activate the Wnt signaling pathway. Altogether, these vectors can probe and modulate the Wnt signaling pathway in experimental settings where persistence of the transgene or gene transfer cannot be accomplished by non-viral techniques.

## Introduction

Lentiviral vectors are powerful tools for gene transfer. Their use ranges from basic to applied research, from *in vitro* to *in vivo* approaches, from overexpression to knock-down, and from embryonic stem cells to transgenic animals (see [1] for a review). Lentiviruses are single-stranded RNA viruses that can infect both dividing and nondividing cells and integrate stably into the host genome to provide a sustained transduction of the target cell. Their safety and efficacy have continuously been improved, most significantly by developing self-inactivating lentiviruses [2] and vesicular stomatitis virus G (VSV-G) protein pseudotyping [3], [4], [5]. The VSV-G protein mediates viral entry through targeting of phospholipids, thus broadening viral tropism to all mammalian cells as well as cells from other vertebrates such as zebrafish and xenopus [6], and allows concentrating viral particles to high titers [7]. Inclusion of a central PolyPurine Tract (cPPT) [8] protects the virus from editing by APOBEC [9] and helps nuclear import of the viral genome [10], and insertion of the Woodchuck hepatitis Post-transcriptional Regulatory Element (WPRE) achieves proper transcript termination through reduction of readthrough transcription [11].

The Wnt signaling pathway is a key player in embryonic development, adult tissue homeostasis, and stem cell maintenance [12], [13], [14]. In the absence of a Wnt signal, β-catenin is phosphorylated by glycogen synthase kinase-3β and targeted for degradation by the proteasome, while transcription factors of the Tcf/Lef family bind to their recognition sequence on the DNA. Wnt signaling leads to the inhibition of β-catenin phosphorylation and allows stabilized β-catenin to accumulate into the nucleus, where it drives gene expression [15]. Wnt target genes switch from a stage of active repression to a stage of active transcription upon triggering of the Wnt signaling pathway. This bimodal state ensures a tight control over Wnt-responsive promoters, and plasmids containing oligomerized Tcf-binding sites [16], [17], [18], [19] have been used extensively to report Wnt signaling. Nevertheless, plasmids can mostly be used in transient experiments and their use is often limited by the efficacy of transfection or electroporation. To circumvent these limitations, Wnt reporter lentiviruses have been used successfully in human embryonal carcinoma cells, mouse embryonic stem cells, embryoid bodies, and *in ovo* infection experiments [20], [21], [22]. Moreover, addition of a drug-resistance or fluorescence cassette has been shown to allow selection of infected cells [16]. In this work, we developed a series of lentiviral vectors to probe and modulate the Wnt signaling pathway, and describe these vectors in the context of existing reporter constructs.

## Results and Discussion

### Selectable Fluorescent Reporter Lentiviruses

**Figure 1:**
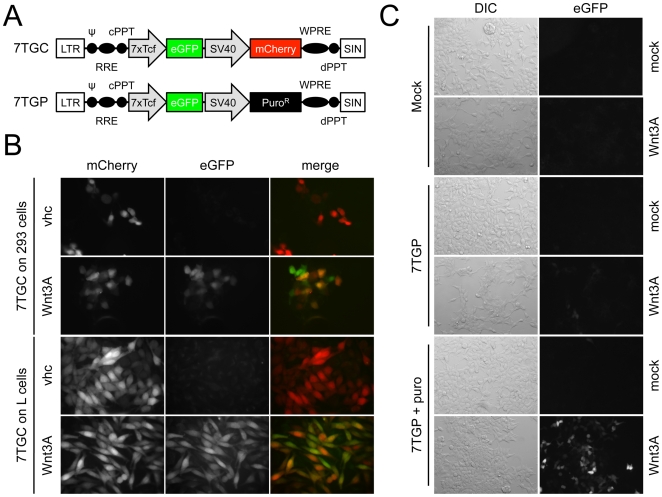
Selectable lentiviruses with Wnt-induced eGFP. A) Schematic description of the 7xTcf-eGFP//SV40-mCherry (7TGC) and 7xTcf-eGFP//SV40-Puro^R^ (7TGP) lentiviruses. LTR: Long Terminal Repeat, ¬: packaging signal, RRE: Rev Response Element, cPPT: central PolyPurine Tract, WPRE: Woodchuck hepatitis Post-transcriptional Regulatory Element, dPPT: distal PolyPurine Tract, SIN: Self Inactivated (LTR). B) HEK293 or mouse L cells were infected with the 7TGC lentivirus. All infected cells expressed mCherry and addition of purified Wnt3A protein (500 ng/ml final, “Wnt3A”) led to expression of eGFP. In absence of Wnt signal (“vhc”), eGFP was not expressed. C) HEK293T cells were infected with the 7TGP lentivirus and either selected in 1 µg/ml puromycin (“7TGP+puro”) or grown in absence of drug (“7TGP”). Expression of eGFP was induced by Wnt3A-conditioned medium (“Wnt3A”) and Wnt-responsive cells were strongly enriched after puromycin.

To develop lentiviral vectors that report on Wnt signaling and allow discrimination between uninfected and non-responding cells, we first inserted an SV40-mCherry [23] selection cassette downstream of the 7xTcf-eGFP reporter cassette [20] (7TGC virus, Fig. 1A, top). In the absence of a Wnt signal (vhc), cells infected with the 7TGC virus displayed only red fluorescence, while addition of Wnt3A protein to the medium induced expression of eGFP (Fig. 1B). Since all infected cells express mCherry, eGFP-negative cells can easily be characterized as either non-responding or uninfected according to their red fluorescence. Moreover, expression of mCherry allows for selection of infected cells by fluorescence activated cell sorting (FACS). When the puromycin resistance gene Puro^R^ was used instead of mCherry (7TGP virus, Fig. 1A, bottom), infected cells could be selected with puromycin to enrich for cells that contained the reporter eGFP cassette (Fig. 1C, compare “7TGP” with “7TGP+puro”). Selection did not enrich for cells with non-specific Wnt reporting activity since eGFP expression was still dependent on the presence of Wnt3A in the medium (compare “mock” with “Wnt3A”). These viruses constitute a substantial improvement over our previous generation of lentiviral reporters that needed indirect approaches such as a control infection with a constitutive eGFP virus [20] or selection of clones [21]. Viruses similar to 7TGC and 7TGP have been described by Biechele and Moon [16], and while the dual-color vector wasn't characterized in sufficient detail to allow comparison with our constructs, the puromycin-selectable, fluorescent reporter vector also conferred robust response to Wnt3A conditioned medium [16].

### Quantitative Measurements and Selection by Survival

**Figure 2:**
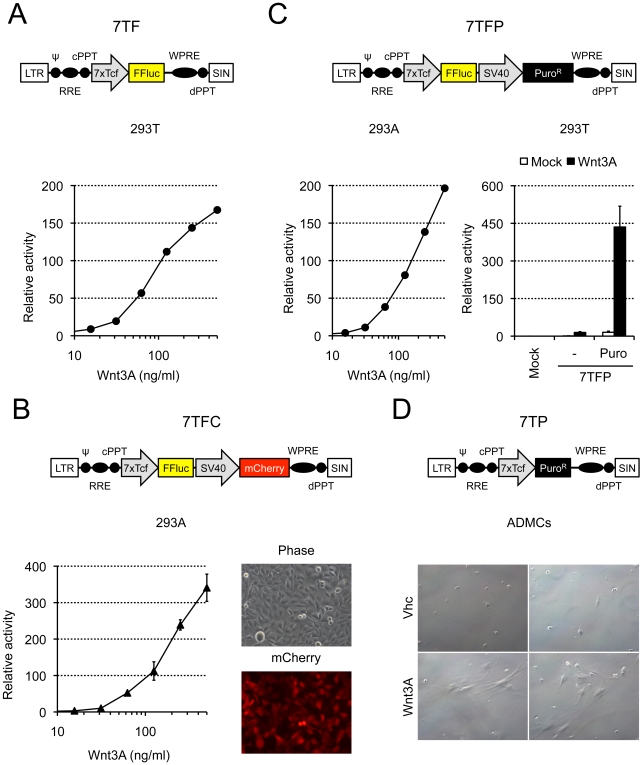
Luminescent and drug-resistant reporters for the Wnt signaling pathway. A) HEK293T cells were infected with the 7xTcf-FFluc (“7TF”) virus and responded to purified Wnt3A protein in a dose-dependent manner. B) HEK293A cells were infected with the 7xTcf-FFluc//SV40-mCherry (“7TFC”) virus and responded similarly to purified Wnt3A protein (left). Cells expressed mCherry in a constitutive manner (right). C) HEK293A cells were infected with the 7xTcf-FFluc//SV40-Puro^R^ (“7TFP”) virus and also responded to purified Wnt3A protein in a dose-dependent manner (left). Selection of 7TFP-infected HEK293T cells with puromycin (1 µg/ml) allowed for enrichment of Wnt-responding cells, resulting in increased luciferase signal upon addition of conditioned medium (right, compare “Puro” with “−”). D) Adipose-derived mesenchymal cells (“ADMCs”) were infected with the 7xTcf-Puro^R^ (7TP) lentivirus. Addition of purified Wnt3A protein (500 ng/ml final, “Wnt3A”) allowed selection of Wnt-responding cells with puromycin (1 µg/ml), while cells incubated in absence of Wnt3A (“vhc”) were all killed by the drug treatment. For all graphs, activity is relative to vehicle- or mock-treated cells.

While fluorescence is the tool of choice for a variety of experiments, it is less appropriate for quantitative measurements. We therefore replaced eGFP with Firefly luciferase (FFluc) to allow quantification of the signal. The 7xTcf-FFluc virus was called 7TF and 293T cells infected with this virus responded to purified Wnt3A in a dose-dependent manner (Fig. 2A). We then added the SV40-mCherry or SV40-Puro^R^ selection cassettes downstream of the 7xTcf-FFluc sequence to allow for fluorescence- or drug-based selection. The 7xTcf-FFluc//SV40-mCherry and 7xTcf-FFluc//SV40-Puro^R^ viruses were called 7TFC (Fig. 2B) and 7TFP (Fig. 2C), respectively. Addition of the constitutive SV40-mCherry or SV40-Puro^R^ cassettes downstream of 7xTcf-FFluc had no influence on the behavior of the reporter, which still responded to purified Wnt3A in a dose-dependent manner (Fig. 2B, left and 2C, left). Similarly to the 7TGC virus (Fig 1B), inclusion of the SV40-mCherry cassette in 7TFC enables selection of the infected cells by FACS because cells constitutively express mCherry (Fig. 2B, right). Addition of the SV40-Puro^R^ cassette allowed for selection of infected cells with puromycin, which resulted in a dramatic increase in the measured signal, (Fig. 2C, right, compare “−” with “puro”). These vectors could be used to determine the response of a given cell population to Wnt signaling, to assess whether cells possess endogenous Wnt activity, or to track activation of the Wnt signaling pathway in live animals. Interestingly, vectors similar to the 7TF and 7TFP viruses have been reported by Biechele and Moon, with experimental data available for the puromycin-selectable vector (pBARLS) [16]. While the pBARLS virus contains 12 Tcf-binding sites, reporter activation by Wnt3A conditioned medium was comparable to that of the 7TFP virus. Although these experiments cannot be strictly compared, this suggests that further increasing the amount of Tcf-binding sites will not significantly increase the sensibility of such reporters. Finally, the 7TFC virus will be particularly useful when drug selection of the infected cells is not possible because of time or survival constraints.

We also created a virus where Puro^R^ expression was driven by the 7xTcf promoter (7xTcf-Puro^R^ virus, 7TP, Fig. 2D). When mouse primary mesenchymal cells infected with the 7TP virus were incubated in puromycin, surviving cells were only detected when the cells had been pre-incubated in medium containing purified Wnt3A (Fig. 2D, compare “Wnt3A” with “vhc”). These viruses could be used to directly isolate cells that respond to exogenous Wnt proteins, cells otherwise activated by Wnt signaling, or cells with intrinsic Wnt activity.

### Modulation of the Wnt Signaling Pathway

**Figure 3:**
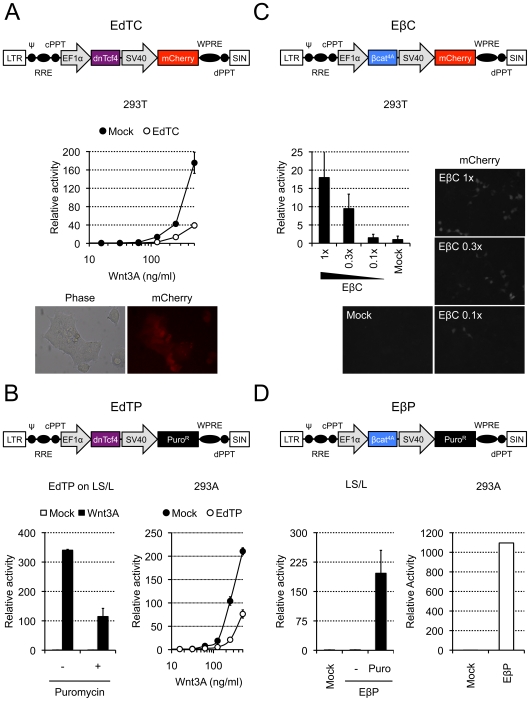
Lentiviruses that inhibit or activate the Wnt signaling pathway. To measure Wnt signaling activity in HEK293T and HEK293A, cells were transfected with the reporter construct pMegaTOPFlash prior to Wnt3A addition. A) HEK293T cells were infected with the hEF1α-dnTcf4//SV40-mCherry (“EdTC”) virus, and mCherry-expressing cells were selected by fluorescence-activated cell sorting. Induction of a Wnt reporter by purified Wnt3A protein (500 ng/ml final) was impaired in EdTC-infected cells compared to mock-infected cells (top). All selected cells were expressing mCherry (bottom). B) Mouse LS/L or HEK293A cells were infected with the hEF1α-dnTcf4//SV40-Puro^R^ (“EdTP”) virus and selected with 1 µg/ml puromycin. Puromycin selection led to enrichment of LS/L cells with an impaired response to Wnt3A-conditioned medium (left, compare “+” with “−” puromycin). Induction of a Wnt reporter by purified Wnt3A protein (500 ng/ml final) was also impaired in EdTP-infected HEK293A cells compared to mock-infected cells (right). C) HEK293T cells were infected with the hEF1α-β-catenin^4A^//SV40-mCherry (“EβC”) virus at different multiplicities of infection (1, 0.3 and 0.1 relative concentration of virus per cell). Increasing the number of infected cells (as seen by the amount of mCherry-positive cells in a confluent field, right) led to increasing activation of the Wnt signaling pathway in absence of any exogenous Wnt source (left). D) Mouse LS/L and HEK293A cells were infected with the hEF1α-β-catenin^4A^//SV40-Puro^R^ (“EβP”) virus and selected with 1 µg/ml puromycin. Selection with puromycin led to the enrichment of cells with constitutive activation of the Wnt signaling pathway (left, compare “−” with “Puro”). Infection of HEK293A with the EβP virus similarly led to ligand-independent activation of the Wnt signaling pathway (right). In panels A and B, activity is relative to vehicle-treated cells. In panels C and D, activity is relative to mock-infected cells.

While the previous viruses report Wnt signaling levels in a variety of settings, activating or blocking the Wnt pathway is often essential in studies on cell proliferation, differentiation and survival. Therefore, we sought to develop vectors to change Wnt signaling strength. To this end, we constructed lentiviruses that express either a dominant negative human Tcf4 (dnTcf4) or an activated form of mouse β-catenin (β-catenin^4A^) from the constitutively active human elongation factor 1α (hEF1α) promoter, together with either the SV40-mCherry or the SV40-Puro^R^ cassette. The hEF1α-dnTCF4//SV40-mCherry, hEF1α-dnTCF4//SV40-Puro^R^, hEF1α-β-catenin^4A^//SV40-mCherry and hEF1α-β-catenin^4A^//SV40-Puro^R^ viruses were called EdTC (Fig. 3A), EdTP (Fig. 3B), EβC (Fig. 3C) and EβP (Fig. 3D), respectively.

LS/L Wnt reporter cells [24] were infected with the EdTC virus, selected by FACS and used in Wnt reporter experiments. As shown in Fig. 3A, cells positive for mCherry (“EdTC”) responded less than mock-infected cells (“Mock”) to purified Wnt3A protein, reflecting the dnTcf4-mediated inhibition of the Wnt signaling pathway. All the sorted cells expressed mCherry (Fig. 3A, bottom). The reporter activity of LS/L cells infected with the EdTP virus was similarly impaired after selection with puromycin (Fig. 3B, bottom left, compare “−” with “+”) and Wnt signaling was equally inhibited in HEK293A cells (Fig. 3B, bottom right, compare “Mock” with “EdTP”). Unlike in transient transfection experiments performed with dnTcf4 (not shown), the EdTC and EdTP viruses did not completely block the Wnt response, probably reflecting a difference in copy numbers between infection and transfection. It is noteworthy that the expression vectors (EdTC, EdTP, EβC and EβP) are produced at lower titers than the other lentiviruses, probably because of their large size [25]. When high titers of these viruses are needed, alternative protocols should be followed (see [26], for an example).

As expected, cells infected with either the EβC or the EβP virus activated the reporter in absence of exogenous Wnt protein (Fig. 3C and 3D). Infection of HEK293T cells with the EβC virus led to an activation of the Wnt signaling pathway that increased with the concentration of lentiviruses used during the infection process (Fig. 3C, compare 1× with 0.3× and 0.1×). Infection of LS/L cells with the EβP virus activated the Wnt reporter after puromycin selection (Fig. 3D, bottom left, compare “−” with “Puro”), an effect similarly observed in HEK293A cells (Fig. 3D, bottom right).

In summary, we have developed a series of lentiviral vectors that report and modulate the Wnt signaling pathway. The key features of our reporter vectors are their ability to allow selection of the infected cells and their different readouts (fluorescence, luminescence and drug-resistance). Some of these vectors (7TGC, 7TGP, 7TF and 7TFP) are comparable in design to previously reported lentiviruses (pBARVef1R, pBARVS, pBARL and pBARLS, respectively) [16], and matching viruses show similar activity, although precise comparison is not possible without performing side-by-side experiments. These similarities underscore the robustness of Wnt reporter lentiviruses, which, added to their versatility, makes them reagents of choice to study activation of the Wnt signaling pathway. Our other luciferase reporter vector (7TFC) allows sorting of infected cells when drug selection is not possible, and the 7TP virus follows a novel strategy to directly isolate cells with high Wnt signaling activity. Altogether, addition of a selection marker enabled efficient cell enrichment, even when the original amount of infected cells was very low (typically less than 5% in Fig. 1C, and probably even less in Fig. 2C and Fig. 3B–D). Our expression vectors can be used to activate or inhibit the Wnt signaling pathway and are similarly selectable. These vectors will be made available to the scientific community through Addgene.

## Methods

### Vectors

Lentiviral vectors have been derived from the lentiviral vectors pCF519 (pLenti hEF1α-MCS//SV40-Puro^R^), pCF566 (pLenti 7xTcf-eGFP, [20]), pCF779 (pLenti hEF1α-MCS//SV40-mCherry), and TOP-dGFP [27].

To construct the 7TP virus (pCF567: pLenti 7xTcf-Puro^R^), the 7xTcf promoter and 5′UTR of pSuperTOPFlash [19] were amplified by PCR with primers oCF236 (CTACTGCAGGGTACCGAGCTCTTACG) and oCF237 (GTCTTCCATGGTGGCTTTAC ) and cut with PstI and NcoI. The puromycin resistance cassette was amplified by PCR from pCF519 using primers oCF238 (TACCATGGGTACCGAGTACAAGCCCACG) and oCF239 (CAATCTTTCACAAATTTTGTAATCCAGAGG) and cut with NcoI and SalI. Both PCR products were ligated into TOP-dGFP cut with PstI and SalI. Of note, while the original pSuperTOPFlash contains eight Tcf-binding sites, only seven were found after plasmid amplification, probably reflecting the unstability of the 8xTcf sequence. To construct the 7TF virus (pCF768: pLenti 7xTcf-Firefly luciferase), Firefly luciferase was isolated from pCF591 (pMegaTOPFlash, [28]) cut with XbaI and inserted into pCF566 cut with XbaI. To construct the 7TGC virus (pCF778: pLenti 7xTcf-eGFP//SV40-mCherry), the 7xTcf-eGFP cassette was first lifted from pCF566 with HpaI and SalI and cloned into pLenti PGK-DsRed (a gift from Laurie Ailles) cut with HpaI and XhoI to yield pCF629. mCherry was amplified from pECE-mCherry (a gift from Julien Sage) using primers oCF399 (ATAAAGCTTACCATGGTGAGCAAGGGCGAGGAG ) and oCF400 (AATAAGCTTGTCGACTTACTTGTACAGCTCGTC), cut with HindIII, and inserted into pRL-SV40 (Promega) cut with HindIII to give pCF767. The SV40-mCherry cassette was then isolated from pCF767 wih BglII (T4-blunted) and SalI and cloned in place of the PGK-DsRed cassette of pCF629 removed by EcoRV and SalI to give 7TGC. pCF519 was cut with SfiI and cloned into pCF778 cut with SfiI to yield 7TGP (pCF846: pLenti 7xTcf-eGFP//SV40-Puro^R^). The SV40-Puro^R^ cassette of pCF519 was isolated with SalI and cloned downstream of Firefly luciferase in pCF768 cut with SalI to yield 7TFP (pCF826: pLenti 7xTcf-Firefly luciferase//SV40-Puro^R^). The SV40-mCherry cassette of pCF780 (see below) was similarly isolated to create 7TFC (pCF829: pLenti 7xTcf-Firefly luciferase//SV40-mCherry). To create EdnTC (pCF780: pLenti hEF1α-dnTcf4//SV40-mCherry), dominant-negative human Tcf4 was amplified from pBSK-DNhTcf4 (obtained from Amanda Mikels) using primers oCF407 (TACAAAGTTAACACCATGGACTACAAAGACGATGAC ) and oCF408 (ATCTAAGCTAGCCTATTCTAAAGACTTGGTGAC ), cut with HpaI and NheI, and inserted into pCF779 cut with EcoRV and XbaI. EdnTP (pCF827: pLenti hEF1α-dnTcf4//SV40-Puro^R^) was created by three-way ligation using pCF519 cut with AvrII and SalI, pCF780 cut with AvrII and NotI, and pCF780 cut with SalI and NotI. Finally, to create EβC (pCF823, pLenti hEF1α-βcatenin^4A^//SV40-mCherry) and EβP (pCF823, pLenti hEF1α-βcatenin^4A^//SV40-Puro^R^), mouse βcatenin^4A^ (S33A, S37A, T41A and S45A, obtained from Amanda Mikels) was amplified with oCF426 (AATAATGTTAACACCATGGCTACTCAAGCTG ) and oCF427 (AATATATCTAGATTACAGGTCAGTATCAAACCAG), cut with HpaI and XbaI, and inserted into pCF519 cut with EcoRV and XbaI or pCF779 cut with EcoRV and XbaI, respectively.

### Lentiviral and Wnt3A Production

Lentiviral production was carried out as described previously [20]. Briefly, ten 10-cm dishes were seeded with 5·10^6^ cells each one day before transfection. For each dish, 10 µg of the lentiviral vector were mixed with 3.5 µg of the VSV- G envelope plasmid and 6.5 µg of the packaging plasmid (pMD2.VSVG and pCMVΔR8.74, respectively [29]). The solution was adjusted to 250 µL with water and mixed with 250 µL 0.5 M CaCl_2_. The precipitate was formed by adding 500 µL of 2xHEPES-buffered saline (280 mM NaCl, 10 mM KCl, 1.5 mM Na2HPO4, 12 mM dextrose, 50 mM HEPES, pH7.2) drop-wise while vortexing and added directly to the cells. The medium was replaced after 16 hours and conditioned twice for 24 hours. The conditioned media were pooled, filtered through a 0.45 µm PES filter, and either used as such or centrifuged at 50,000g for 2 hours 20 minutes. After centrifugation, the viral pellet was resuspended in 400 µL 0.1% BSA in PBS. Wnt3A proteins were purified by affinity purification and size fractionation as described previously [30]. The vehicle control was obtained by diluting the size fractionation buffer accordingly in complete medium. When conditioned media were used, an equivalent number of HEK293 cells or HEK293 cells expressing Wnt3A were seeded, media were conditioned for three to four days, collected, spun at 300g for 5 minutes and filtered through a 0.45 µM filter.

### Wnt Reporter Assays

Reporter assays have been described earlier [30]. While LS/L cells [24] had been stably transfected with pSuperTOPFlash and pEF1/Myc-His/LacZ (Invitrogen), HEK293A and HEK293T cells were transfected with pMegaTOPFlash [28] and pEF1/Myc-His/LacZ or pEF1α-LacZ-IRES-Puro^R^ one day prior to the addition of Wnt3A. Reporter assays were performed with the dual-light combined reporter gene assay system (Applied Biosystems) with a Centro LB960 luminometer (Berthold) according to the manufacturer's instructions. β-galactosidase activity was used to normalize the values, which are represented with their standard deviation.
